# Divergent Blood Pressure Thresholds in Hypertensive Disorders of Pregnancy: A Narrative Review of Global Guideline Discordance and Clinical Implications

**DOI:** 10.1155/ijhy/8873687

**Published:** 2026-07-02

**Authors:** Prosper Akankwasa, Jackson Kakooza, Esther Namutosi, John Katongole, Hakizimana Theoneste, Ephraim Onaba, Fatima Abdirizak Muse, Carlos Antonio Batista Cedeño, Catherine R. Lewis, Emmanuel Okurut

**Affiliations:** ^1^ Department of Obstetrics and Gynecology, Kampala International University, Western Campus Ishaka, Bushenyi, Uganda, kiu.ac.ug; ^2^ Department of Surgery, Kampala International University, Western Campus Ishaka, Bushenyi, Uganda, kiu.ac.ug; ^3^ Department of Surgery, St. Joseph’s Hospital Kitovu, Masaka, Uganda

**Keywords:** blood pressure thresholds, global maternal health, guideline variation, hypertensive disorders of pregnancy, preeclampsia, treatment initiation

## Abstract

Hypertensive disorders of pregnancy (HDP) remain a leading cause of preventable maternal and perinatal morbidity and mortality. Although blood pressure (BP) thresholds guide diagnosis, treatment, and delivery, international and national guidelines differ, resulting in inconsistent management. This narrative review synthesizes contemporary evidence to map divergences in BP thresholds across global guidelines and evaluate their implications for clinical decision‐making and maternal and fetal outcomes, particularly in low‐ and middle‐income countries (LMICs). Comprehensive searches of PubMed, Scopus, Web of Science, and Lens.org were performed. Twenty‐five eligible sources, including 12 major international and national guidelines and 13 supporting peer‐reviewed sources, were synthesized following SANRA principles. Additional contextual references were cited where necessary but were not counted as included sources in the threshold‐synthesis dataset. Diagnostic thresholds, hypertension criteria, treatment initiation thresholds, target BP goals, and magnesium sulfate indications were reviewed. All 12 guidelines uniformly diagnosed hypertension at ≥ 140/90 mmHg. Near‐consensus exists for defining severe hypertension at ≥ 160/110 mmHg, though several LMIC guidelines adopt lower thresholds (≥ 150/100–110 mmHg). The most clinically impactful divergence concerns antihypertensive treatment initiation, which varies from ≥ 140/90 mmHg to ≥ 160/110 mmHg, with intermediate thresholds (≥ 150/100 mmHg) used in several LMICs. Additional differences such as the inclusion of fetal growth restriction in some diagnostic frameworks further contribute to misclassification. These inconsistencies create a 20 mmHg “treatment gap,” leading to guideline‐dependent over‐ or under‐treatment and widening disparities in resource‐limited settings. Marked threshold discord across the 12 major HDP guidelines results in systematic misclassification and divergent management pathways. Harmonization initiatives, transparent communication of evidence limitations, and context‐specific implementation research, particularly in LMICs, are urgently needed to support equitable, evidence‐based maternal care.

## 1. Introduction

Hypertensive disorders of pregnancy (HDP), encompassing chronic hypertension, gestational hypertension, preeclampsia, eclampsia, and preeclampsia superimposed on chronic hypertension, represent a critical public health concern worldwide [1–3]. These conditions complicate approximately 5%–10% of all pregnancies globally, standing as a leading cause of maternal, fetal, and neonatal morbidity and mortality [4–6].

The burden is disproportionately severe in low‐ and middle‐income countries (LMICs), which account for 99% of global maternal deaths, with complications from HDP ranking among the foremost preventable causes of severe maternal and perinatal morbidity and mortality [7, 8]. Preeclampsia and eclampsia are the second most common direct causes of maternal deaths worldwide, contributing to roughly 16% of maternal fatalities in LMICs, where preeclampsia is often the first or second leading avoidable cause of death [7, 9].

In Sub‐Saharan Africa, exemplified by Nigeria, the pooled prevalence of preeclampsia and eclampsia reaches 6.04%, far exceeding rates in high‐income countries, and is linked to high maternal mortality alongside fetal mortality rates as high as 16.73% [10]. Compounding this, the rising prevalence of chronic hypertension driven by factors such as obesity and advanced maternal age heightens risks of superimposed preeclampsia, preterm delivery, low birth weight, and perinatal death [1].

Blood pressure (BP) thresholds serve as the foundational cornerstone for the diagnosis, classification, and management of HDP, directly influencing diagnostic pathways, risk stratification, treatment escalation, and clinical outcomes [4]. Conventionally defined as a systolic BP (SBP) ≥ 140 mmHg and/or diastolic BP (DBP) ≥ 90 mmHg on two separate occasions, these thresholds guide essential decisions, including the timing of antihypertensive therapy to prevent progression to severe hypertension and associated complications, magnesium sulfate administration for eclampsia prophylaxis in cases of severe BP elevations (SBP ≥ 160 mmHg or DBP ≥ 110 mmHg) with preeclampsia, and delivery timing based on severity classification and gestational age [1, 3]. Accurate application ensures timely recognition of severity, enabling interventions that mitigate risks such as acute kidney injury, heart failure, stroke, fetal growth restriction (FGR), preterm birth, stillbirth, and neonatal intensive care unit admission [1, 4, 10].

However, a pervasive lack of consensus and variation in diagnostic and treatment BP thresholds across influential international and national clinical practice guidelines (CPGs), including those from the American College of Obstetricians and Gynecologists (ACOG), International Society for the Study of Hypertension in Pregnancy (ISSHP), National Institute for Health and Care Excellence (NICE), and World Health Organization (WHO), creates profound clinical confusion, misclassification of disease severity, and inconsistent decision‐making [3, 4, 7, 11]. This discordance arises from conflicting interpretations of evidence, such as the applicability of data from trials like Chronic Hypertension and Pregnancy (CHAP) [12] to nonchronic HDP forms, and manifests in divergent recommendations. For instance, ACOG advocates antihypertensive initiation primarily at severe levels (≥ 160/110 mmHg) except in chronic cases, while ISSHP, NICE, and WHO support treatment at lower thresholds (≥ 140/90 mmHg) for any HDP, with further stratification for moderate hypertension (150–159/100–109 mmHg) in some guidelines [3, 11, 13]. Such inconsistencies lead to system‐level quality‐of‐care gaps, including diagnostic drift (e.g., classifying cases as gestational hypertension under ACOG but preeclampsia under NICE or ISSHP due to inclusion of FGR), over‐ or under‐treatment, and variable target BP goals (e.g., < 135/85 mmHg in some versus < 150/100 mmHg in others), all exacerbated in fragmented settings where multiple guidelines circulate [4, 7, 11, 14, 15].

From a quality improvement (QI) perspective, this guideline discordance constitutes a major safety gap, hindering equitable care and contributing to preventable maternal and neonatal morbidity and mortality, including acute complications like stroke, cardiomyopathy, and organ failure, as well as postpartum deaths [3, 4]. In resource‐constrained environments like Sub‐Saharan Africa, where provider training varies, resources are limited, and adherence to antenatal follow‐up is poor, these inconsistencies amplify disparities, resulting in delayed recognition of severity, inappropriate interventions, and heightened risks of undetected hypertension progression [16, 17]. The failure to harmonize thresholds undermines standardized clinical practice, perpetuating clinical confusion and variations in care quality that impede timely diagnosis and management [7].

In this narrative review, we aim to systematically map these divergences in BP cutoffs for defining hypertension, severe hypertension, and severe features of preeclampsia across major guidelines, synthesizing recent evidence to elucidate their clinical and QI implications, particularly in low‐resource settings. By addressing this knowledge gap, this review paves the way for actionable strategies to harmonize thresholds and protocols, enhancing the generalizability and feasibility of HDP care.

## 2. Methods

### 2.1. Study Design and Rationale

This study was conducted as a narrative review with a predefined conceptual framework to synthesize divergent BP thresholds and management recommendations across major international and national CPGs for HDP. A narrative approach was selected because the included evidence was heterogeneous, comprising guidelines, observational studies, and implementation reports precluding quantitative synthesis while requiring interpretive integration. Methodological conduct and reporting followed the Scale for the Quality Assessment of Narrative Reviews (SANRA) criteria [18] to ensure scientific rigor.

### 2.2. Search Strategy

A comprehensive search of peer‐reviewed literature and authoritative guideline repositories was conducted in July 2025 across the following databases: PubMed (MEDLINE), Scopus, Web of Science, and Lens.org. Guideline searches included WHO, ACOG, International Federation of Obstetrics and Gynaecology (FIGO), Royal College of Obstetricians and Gynaecologists (RCOG), NICE, ISSHP, Society of Obstetricians and Gynaecologists of Canada (SOGC), and national ministries of health from Uganda, Kenya, Nigeria, South Africa, and Ghana.

Search terms combined three domains using Boolean operators (AND/OR):

• HDP terms: “hypertensive disorders of pregnancy,” “preeclampsia,” “gestational hypertension,” “eclampsia,” “superimposed preeclampsia”
• BP measurement terms: “blood pressure thresholds,” “severe hypertension,” “antihypertensive initiation,” “target BP”
• Guideline terms: “clinical practice guideline,” “recommendation,” “ACOG,” “ISSHP,” “NICE,” “WHO,” “protocol”

Searches were restricted to English‐language publications published between January 2020 and July 2025 to capture contemporary evidence and guideline updates. Forward and backward citation chaining was performed for all included sources.

### 2.3. Inclusion and Exclusion Criteria

CPGs, systematic reviews, observational studies, or implementation studies were included. Publications addressing BP thresholds for HDP diagnosis, severe hypertension, antihypertensive initiation, magnesium sulfate indications, target BP goals, and delivery timing were included. All studies published addressing the previously mentioned criteria and evidence concerning pregnant or postpartum women were also included. Only studies with full text available and published between January 2020 and July 2025 were included. Guidelines published prior to 2020 (unless still active, e.g., WHO 2011), editorials, commentaries, and case reports were excluded. Reviews on topics not related to HDP and non‐English manuscripts were also excluded.

### 2.4. Study Selection Process

Literature search results were imported into EndNote X20, and duplicates were removed using automated and manual methods. Two independent reviewers screened titles and abstracts against inclusion/exclusion criteria. Discrepancies were resolved through discussion or third‐reviewer consultation. The selection process followed a modified PRISMA 2020 flow diagram adapted for narrative reviews [19].

### 2.5. Quality and Rigor Assessment

To enhance methodological credibility, the 12 included guidelines were appraised descriptively using selected AGREE II domains, particularly scope and purpose, rigor of development, clarity of presentation, applicability, and editorial independence [20]. Systematic reviews were assessed using key principles from AMSTAR 2 [21]. Primary observational studies were evaluated using methodological criteria including design adequacy, confounding control, and completeness of outcomes. These appraisals were used to contextualize the transparency, applicability, and evidentiary strength of each source during synthesis; they were not used as exclusion criteria because the objective was to map guideline discordance currently encountered in clinical practice.

### 2.6. Data Extraction and Synthesis

A structured extraction framework captured the following: BP thresholds for diagnosis, severe hypertension, treatment initiation; severe‐feature definitions; magnesium sulfate indications; target BP goals; delivery timing; measurement techniques; classification differences across guidelines; and contextual implementation barriers (especially in LMICs).

Data were synthesized thematically and aligned with the review objectives. Comparative mapping tables were developed to illustrate convergence and divergence across guidelines. Findings were integrated narratively, emphasizing clinical implications, misclassification risks, and relevance to low‐resource health systems.

## 3. Results

### 3.1. Study Selection

**FIGURE 1:**
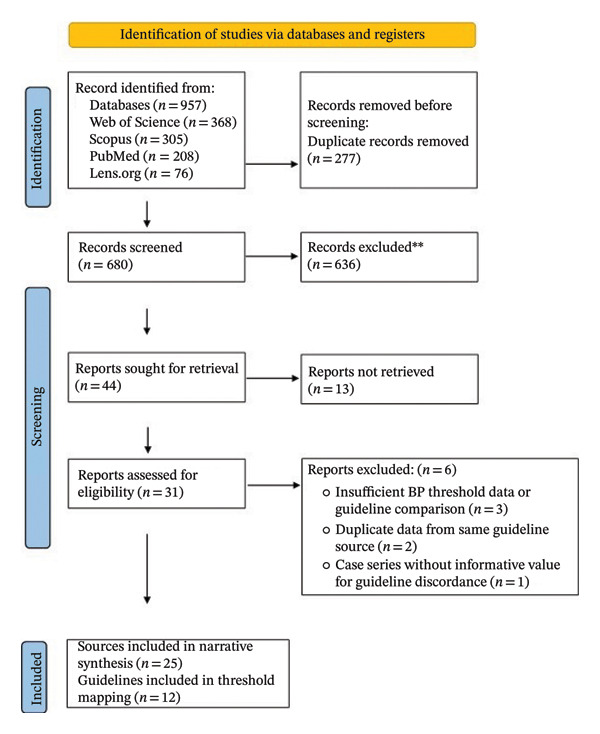
PRISMA flow diagram demonstrating the article screening and selection.

A total of 957 records were identified through database searches, including Web of Science (*n* = 368), Scopus (*n* = 305), PubMed (*n* = 208), and Lens.org (*n* = 76). After removal of 277 duplicate records, 680 records remained for title and abstract screening. Of these, 636 records were excluded because they did not address BP thresholds or HDP classification (*n* = 412), were published before 2020 and were not active guidelines (*n* = 98), were non‐English publications (*n* = 54), were conference abstracts, editorials, or commentaries (*n* = 47), or were unrelated to HDP (*n* = 25). Forty‐four reports were sought for retrieval, of which 13 could not be retrieved because full text was unavailable. The remaining 31 reports underwent full‐text assessment. Six reports were excluded because of insufficient BP‐threshold or guideline‐comparison data (*n* = 3), duplication of information from the same guideline source (*n* = 2), or case‐series design without meaningful relevance to guideline discordance (*n* = 1). Ultimately, 25 eligible sources were included in the final narrative synthesis, including 12 major international and national guidelines (Figure 1).

### 3.2. Guideline Quality Appraisal Summary

Descriptive AGREE II appraisal showed that the 12 included guidelines were generally strongest in scope, purpose, and clarity of presentation. Most guidelines clearly defined HDP categories, diagnostic BP thresholds, severe hypertension thresholds, magnesium sulfate indications, and delivery‐timing recommendations. However, methodological transparency varied substantially. International and high‐income‐country guidelines, including NICE, ISSHP, ACOG, SOGC, and FIGO, generally provided clearer links between recommendations and supporting evidence, whereas several national or LMIC guidelines were more pragmatic but provided limited detail on evidence‐search methods, grading of recommendations, editorial independence, implementation tools, audit indicators, and resource implications. Applicability was especially variable: LMIC guidelines were often more context‐sensitive but less explicit about implementation monitoring. These appraisal findings were considered during interpretation of discordant recommendations, but no guideline was excluded on the basis of quality appraisal.

### 3.3. Patterns of BP Thresholds Across Guidelines

Analysis of 12 major international and national guidelines revealed three consistent narrative patterns: areas of strong consensus, moderate variation, and substantial divergence in BP thresholds used for diagnosis, severity classification, treatment initiation, and timing of delivery. Most guidelines, including ACOG, NICE, ISSHP, WHO, SOGC, FIGO, and the national guidelines of Uganda, South Africa, Kenya, Nigeria, and Ghana, uniformly diagnose hypertension in pregnancy at ≥ 140/90 mmHg. This area showed near‐universal agreement, as reflected in Table 1.

**TABLE 1:** Blood pressure thresholds for hypertensive disorders of pregnancy management.

Guideline	HTN diagnosis	Severe HTN	Treatment initiation	Target BP	MgSO4 threshold	Delivery timing
High‐income country guidelines						
ACOG (2020)	≥ 140/90 mmHg	≥ 160/110 mmHg	≥ 160/110 mmHg (acute, GH/PE)	< 160/110 mmHg	Severe features OR ≥ 160/110 mmHg	≥ 37w (not severe); ≥ 34w (severe)
NICE (2023)	≥ 140/90 mmHg	≥ 160/110 mmHg	≥ 140/90 mmHg (any HDP)	≤ 135/85 mmHg	Severe preeclampsia	≥ 37w (not severe); earlier if severe
ISSHP (2022)	≥ 140/90 mmHg	≥ 160/110 mmHg	≥ 140/90 mmHg	110–140/< 85 mmHg	Severe features present	≥ 37w (not severe); ≥ 34w (severe)
SOGC (2022)	≥ 140/90 mmHg	≥ 160/110 mmHg	≥ 140/90 mmHg	≤ 135/85 mmHg	Severe preeclampsia	≥ 37w (not severe); ≥ 34w (severe)
FIGO (2019)	≥ 140/90 mmHg	≥ 160/110 mmHg	≥ 140/90 mmHg	Not specified	Severe features present	≥ 37w (not severe); ≥ 34w (severe)
LMIC and international guidelines						
WHO (2011/2020)	≥ 140/90 mmHg	≥ 160/110 mmHg	≥ 150/100–110 mmHg	Above low‐normal	BP ≥ 160/110 OR severe PE	Immediate if severe; ≥ 37w if mild
Uganda MoH (2024)	≥ 140/90 mmHg	≥ 150/100–110 mmHg	≥ 150/95–100 mmHg	Not specified	BP ≥ 150/100–110 OR severe	Immediate if severe; ≥ 37w if nonsevere
Nigeria SOGON (2022)	≥ 140/90 mmHg	≥ 150/100–110 mmHg	≥ 150/100 mmHg	Not specified	BP ≥ 150/100–110 OR severe	Immediate if severe; ≥ 37w if nonsevere
Kenya MoH (2023)	≥ 140/90 mmHg	≥ 160/110 mmHg	≥ 150/100 mmHg	Not specified	BP ≥ 150/100–110	Immediate if severe; ≥ 37w if mild
Ghana GHS (2022)	≥ 140/90 mmHg	≥ 150/100–110 mmHg	≥ 150/100 mmHg	Not specified	BP ≥ 150/100–110 OR severe	Immediate if severe; ≥ 37w if mild
SOGP Pakistan (2022)	≥ 140/90 mmHg	≥ 160/110 mmHg	> 140/90 mmHg	130/85 mmHg	BP ≥ 160/110 OR severe PE	≥ 37w (not severe); earlier if severe
South Africa DoH (2019)	≥ 140/90 mmHg	≥ 150/100–110 mmHg	≥ 150/100 mmHg	Not specified	BP ≥ 150/100–110 OR severe	Immediate if severe; ≥ 37w if mild

*Note:* FIGO, International Federation of Obstetrics and Gynaecology; HTN, hypertension; MgSO_4_, magnesium sulfate; NICE, National Institute for Health and Care Excellence; PE, preeclampsia.

Abbreviations: ACOG, American College of Obstetricians and Gynecologists; BP, blood pressure; DoH, Department of Health; GH, gestational hypertension; GHS, Ghana Health Service; HDP, hypertensive disorders of pregnancy; ISSHP, International Society for the Study of Hypertension in Pregnancy; LMIC, low‐ and middle‐income countries; MoH, Ministry of Health; SOGON, Society of Gynaecology and Obstetrics of Nigeria; SOGP, Society of Obstetricians and Gynaecologists of Pakistan; WHO, World Health Organization.

In contrast, severe hypertension thresholds demonstrate moderate variation. While most guidelines apply ≥ 160/110 mmHg, several LMIC guidelines adopt a slightly lower severe‐range diastolic threshold (≥ 100–110 mmHg) [22–24], implying earlier intervention compared with high‐income country guidelines [2, 13].

The greatest divergence occurred in antihypertensive treatment initiation thresholds. ACOG reserves acute oral or IV therapy for ≥ 160/110 mmHg in gestational hypertension or preeclampsia, whereas NICE, ISSHP, SOGC, and FIGO recommend treatment beginning at ≥ 140/90 mmHg for any hypertensive disorder [4, 11]. LMIC guidelines generally adopt an intermediate position, initiating treatment at ≥ 150/100 mmHg, consistent with WHO’s older or modified recommendations [2, 23].

Despite substantial variation in treatment thresholds, delivery timing recommendations were consistent across guidelines: ≥ 37 weeks for hypertension or preeclampsia without severe features and ≥ 34 weeks or earlier when severe features are present [11, 25, 26].

### 3.4. Diagnostic Threshold: Universal Agreement

Analysis revealed unanimous consensus across all 12 guidelines analyzed which uniformly diagnose hypertension in pregnancy at ≥ 140/90 mmHg. This represents the single point of complete international harmonization. ISSHP provided the most detailed measurement specifications, requiring office BP ≥ 140/90 mmHg on ≥ 2 occasions ≥ 4 h apart or, alternatively, home BP ≥ 135/85 mmHg when ambulatory monitoring is utilized [3]. The consistency of this threshold across diverse healthcare contexts suggests robust evidence consensus and practical feasibility at this BP level.

### 3.5. Severe Hypertension Threshold: Near‐Consensus With Regional Variations

Near‐consensus emerged regarding the threshold for severe hypertension, with notable regional adaptations. Eight of 12 analyzed guidelines consistently defined severe hypertension as ≥ 160/110 mmHg. In contrast, guidelines from four LMICs adopted a lower threshold of ≥ 150/100–110 mmHg. This 10 mmHg systolic reduction reflects pragmatic adaptation to resource‐limited contexts where delayed presentation and limited acute‐care capacity necessitate earlier escalation of care intensity.

### 3.6. Treatment Initiation Threshold: Major Divergence

The most substantial and clinically consequential divergence emerged in antihypertensive treatment initiation thresholds, creating three distinct management paradigms. The conservative approach, exemplified by ACOG, reserves acute antihypertensive therapy for BP ≥ 160/110 mmHg in women with gestational hypertension or preeclampsia, while paradoxically recommending treatment at ≥ 140/90 mmHg for chronic hypertension, creating a dual‐threshold system within a single guideline [3, 11].

The liberal intervention approach adopted by NICE, ISSHP, SOGC, SOGP, and contemporary WHO guidance advocates treatment initiation at ≥ 140/90 mmHg for any HDP [2–4]. NICE and ISSHP specify treatment at ≥ 140/90 mmHg if comorbidities are present or ≥ 150/100 mmHg otherwise [3, 4, 11] FIGO did not specify guidelines for treatment initiation.

LMIC guidelines adopted an intermediate position, establishing ≥ 150/100 mmHg as the treatment threshold [13, 22, 23, 27]. This 20 mmHg treatment gap between the most conservative (ACOG:160 mmHg) and most aggressive (NICE/ISSHP: 140 mmHg) approaches represents a clinically significant window during which one guideline mandates immediate pharmacological intervention while another prescribes only observation.

### 3.7. Magnesium Sulfate Initiation Threshold

Magnesium sulfate is indicated for severe features or eclampsia across ACOG, NICE, WHO, ISSHP, FIGO, SOGC, Uganda MoH, South Africa DoH, Nigeria SOGON, Kenya MoH, and Ghana GHS [11, 28]. ACOG requires it for acute‐onset severe hypertension (≥ 160/110 mmHg) [11, 25]. The WHO has guidelines for prevention and treatment of eclampsia [11]. SOGP indicates magnesium sulfate for severe preeclampsia features or when BP ≥ 160/110 mmHg with signs of impending eclampsia [2]. Thresholds are tied to severe BP ≥ 160/110 mmHg or eclampsia.

### 3.8. Delivery Timing Recommendations Linked to Thresholds

Delivery is recommended at ≥ 37 weeks without severe features and ≥ 34 weeks with severe features across ACOG, NICE, WHO, ISSHP, FIGO, SOGC, Uganda MoH, South Africa DoH, Nigeria SOGON, Kenya MoH, and Ghana GHS [11, 25, 26]. ACOG requires delivery if uncontrolled severe BP occurs [11, 25]. SOGP indicates delivery at ≥ 37 weeks for GH/PE or earlier for compromise like severe uncontrolled HTN [2]. Expectant management is optional for nonsevere but contraindicated for severe.

### 3.9. Differences in Definitions of HDP Across Guidelines

Guidelines exhibit variability in defining HDP categories, contributing to misclassification and clinical confusion. Chronic hypertension is uniformly defined across guidelines as hypertension present before pregnancy or diagnosed before 20 weeks’ gestation, with a threshold of ≥ 140/90 mmHg. ISSHP specifies that for de novo diagnoses made during pregnancy, documentation of elevated BP before 20 weeks’ gestation distinguishes chronic hypertension from gestational hypertension [15]. NICE, WHO, and ACOG align on this pre‐20‐week cutoff [1, 4, 7]. Guidelines show consensus on this definition with no clinically significant variations in diagnostic criteria beyond the gestational age timing.

Gestational hypertension is consistently defined across all guidelines as new‐onset hypertension ≥ 140/90 mmHg occurring after 20 weeks’ gestation in the absence of proteinuria or organ dysfunction [2, 3, 7, 11]. While modern guidelines have largely abandoned the historical “30/15 rule” (30 mmHg systolic or 15 mmHg diastolic rise from baseline), some clinicians may still consider such elevations as warranting closer monitoring when combined with other clinical features, though this is not included in contemporary diagnostic criteria [11]. According to ACOG, preeclampsia is hypertension with proteinuria or end‐organ effects [3, 11, 14]. NICE, ISSHP, FIGO, and SOGC broaden this diagnosis to include maternal organ dysfunction, acute kidney injury, liver dysfunction, neurological features, hemolysis, thrombocytopenia, or FGR/uteroplacental dysfunction without mandatory proteinuria [7, 14, 15, 26]. The WHO historically hinges on proteinuria but broadens to include organ dysfunction [7]. SOGP requires either proteinuria or maternal end‐organ dysfunction for preeclampsia diagnosis, but states proteinuria is not essential if organ dysfunction is present [2]. All guidelines define eclampsia as seizures in a preeclampsia patient. Superimposed preeclampsia is defined as new preeclampsia signs in chronic hypertension after 20 weeks [1, 4].

ACOG defines hypertension with severe features as BP ≥ 160/110 mmHg with platelets < 100,000/μL, impaired liver function, renal insufficiency, pulmonary edema, or cerebral/visual symptoms [11, 14, 25]. NICE, WHO, ISSHP, FIGO, SOGC, Uganda MoH, South Africa DoH, Nigeria SOGON, Kenya MoH, and Ghana GHS include BP ≥ 160/110 mmHg, organ dysfunction, presence of symptoms, or FGR [3, 7]. SOGP includes thrombocytopenia, elevated liver enzymes, renal deterioration, neurological symptoms, pulmonary edema, and HELLP syndrome [2].

### 3.10. Non‐BP Diagnostic Criteria: A Second Layer of Discordance

Beyond BP thresholds, guidelines diverge substantially in nonhypertensive criteria used to diagnose preeclampsia and define severe features. These differences compound threshold‐based misclassification by creating additional diagnostic branch points where guideline choice determines disease categorization. Table 2 systematically compares laboratory and clinical criteria across all examined guidelines, revealing three critical areas of divergence: proteinuria requirements, acceptance of FGR as standalone criterion, and quantitative thresholds for organ dysfunction markers.

**TABLE 2:** Nonblood pressure diagnostic criteria for preeclampsia across guidelines.

Guideline	Proteinuria required?	FGR sufficient criterion?	Platelet threshold	Liver enzyme threshold	Renal dysfunction threshold	Other maternal organ dysfunction criteria
ACOG (2020)	No (if organ dysfunction present)	No (must have other organ dysfunction)	< 100,000/μL	ALT or AST ≥ 2 × ULN	Serum creatinine > 1.1 mg/dL OR doubling baseline	Pulmonary edema; new‐onset cerebral/visual symptoms
NICE (2023)	No	Yes (with HTN ≥ 20 wks)	< 150,000/μL	Not specified (impaired liver function)	Progressive renal insufficiency (falling eGFR)	Neurological features; hemolysis
ISSHP (2022)	No	Yes (with HTN ≥ 20 wks)	< 100,000/μL	ALT > 40 IU/L	Serum creatinine ≥ 90 μmol/L OR oliguria < 80 mL/4h	Neurological features; pulmonary edema
FIGO (2019)	No	Yes (with HTN ≥ 20 wks)	< 100,000/μL	Elevated transaminases (≥ 2 × normal)	Renal insufficiency (creatinine elevation)	Pulmonary edema; neurological symptoms; hemolysis
SOGC (2022)	No	Yes (with HTN ≥ 20 wks)	< 100,000/μL	≥ 2 × normal or > 70 IU/L	Serum creatinine > 90 μmol/L	Neurological symptoms; pulmonary edema
WHO (2011/2020)	Yes (traditionally)	Variable (not standalone)	< 100,000/μL	Not specified	Not specified	Pulmonary edema; maternal organ dysfunction
Uganda MoH (2024)	Yes (HTN + proteinuria)	No (not standalone)	< 100,000/μL	AST/ALT ≥ 2 × ULN	Serum creatinine ≥ 1.1 mg/dL	Pulmonary edema; persistent cerebral/visual symptoms
Nigeria SOGON (2022)	Yes	No	< 100,000/μL	Elevated transaminases	Renal impairment	Neurological symptoms; pulmonary edema
Kenya MoH (2023)	Yes	Not specified	< 100,000/μL	Elevated liver enzymes	Renal dysfunction	Not detailed
Ghana GHS (2022)	Yes	No	< 100,000/μL	Elevated enzymes	Renal dysfunction	Not detailed
South Africa DoH (2019)	Yes	No	< 100,000/μL	Elevated transaminases	Renal insufficiency	Pulmonary edema; neurological symptoms
SOGP Pakistan (2022)	Yes	No	< 100,000/μL	≥ 2 × normal (transaminases)	Serum creatinine > 1.1 mg/dL	Pulmonary edema; neurological symptoms; HELLP

*Note:* ALT, alanine aminotransferase; AST, aspartate aminotransferase; FIGO, International Federation of Obstetrics and Gynaecology; HTN, hypertension; NICE, National Institute for Health and Care Excellence.

Abbreviations: ACOG, American College of Obstetricians and Gynecologists; BP, blood pressure; DoH, Department of Health; eGFR, estimated glomerular filtration rate; FGR, fetal growth restriction; GHS, Ghana Health Service; ISSHP, International Society for the Study of Hypertension in Pregnancy; MoH, Ministry of Health; SOGON, Society of Gynaecology and Obstetrics of Nigeria; SOGP, Society of Obstetricians and Gynaecologists of Pakistan; ULN, upper limit of normal; WHO, World Health Organization.

### 3.11. Divergent Diagnostic Criteria and Disease Reclassification

The inclusion or exclusion of specific diagnostic criteria across guidelines creates systematic misclassification that extends beyond BP thresholds alone. The most striking example involves uteroplacental dysfunction as a diagnostic criterion for preeclampsia. Contemporary guidelines including NICE, ISSHP, FIGO, and SOGC explicitly recognize FGR or abnormal umbilical artery Doppler findings in the presence of new‐onset hypertension (≥ 140/90 mmHg) after 20 weeks’ gestation as sufficient for preeclampsia diagnosis, even in the complete absence of maternal proteinuria or biochemical evidence of organ dysfunction [7, 14, 15].

ACOG guidelines, however, do not recognize isolated uteroplacental dysfunction as a preeclampsia criterion. Under ACOG classification, this identical clinical presentation of new hypertension with FGR but without proteinuria or maternal organ involvement would be categorized as gestational hypertension complicated by FGR, not preeclampsia [11]. In contrast, under NICE/ISSHP criteria, the same patient would receive a preeclampsia diagnosis based on the presence of uteroplacental dysfunction [7, 14, 15].

Beyond categorical diagnosis, quantitative thresholds for laboratory markers of organ dysfunction introduce additional classification variability. Guidelines variously define clinically significant thrombocytopenia as platelet counts < 100,000/μL (ACOG, most LMIC guidelines) versus < 150,000/μL (some ISSHP interpretations) and hepatic dysfunction as transaminase elevation ≥ 2 × upper limit of normal versus absolute values > 40–70 IU/L depending on institutional reference ranges [7, 14, 15]. These seemingly technical distinctions determine whether a patient crosses the threshold from preeclampsia without severe features to preeclampsia with severe features, a classification that fundamentally alters delivery timing, monitoring intensity, and magnesium sulfate administration decisions.

### 3.12. Over‐Treatment and Under‐Treatment

Discordant antihypertensive initiation thresholds produce both over‐treatment and under‐treatment depending on the guideline followed. Guidelines recommending treatment at ≥ 140/90 mmHg may lead to earlier medication exposure and theoretical risk of impaired uteroplacental perfusion, whereas ACOG’s higher acute‐treatment threshold of ≥ 160/110 mmHg for gestational hypertension and preeclampsia (but ≥ 140/90 mmHg for chronic hypertension) may delay intervention and increase the risk of progression to severe hypertension, stroke, or organ damage [3, 4, 11]. Aggressive target BP goals (e.g., DBP < 85 mmHg in ISSHP and SOGC) carry potential risk of iatrogenic FGR, while conservative targets may leave women exposed to uncontrolled severe‐range BP [3, 4].

### 3.13. Measurement Variability

Guidelines exhibit minor but relevant differences in recommended BP measurement techniques. Most require a second confirmatory reading within 4 h [7]. ACOG specifies the sitting or left lateral recumbent position during labor, cuff at heart level, and Korotkoff phase V for DBP [1, 11]. NICE and others mandate 5‐min rest, sitting position, arm at heart level, and appropriate cuff size [7, 29]. Automated devices are recommended by ISSHP, FIGO, and SOGC, with validation in pregnancy emphasized [7]. In low‐resource contexts, aneroid devices are permitted when calibrated mercury or automated devices are unavailable [2].

## 4. Discussion

### 4.1. Principal Findings and Clinical Implications

This systematic analysis of 12 major international and national guidelines governing HDP revealed a paradox: universal consensus on the diagnostic threshold (≥ 140/90 mmHg) coexists with profound divergence in treatment thresholds, target BP goals, and criteria defining severe disease. While this diagnostic agreement might appear reassuring, our findings demonstrate that subsequent management pathways diverge so substantially that guideline selection, rather than patient‐specific clinical factors, becomes the primary determinant of whether a pregnant woman with elevated BP receives antihypertensive medication, how aggressively that BP is lowered, when magnesium sulfate prophylaxis is initiated, and at what gestational age delivery is recommended. These are not trivial variations in clinical nuance; they represent fundamentally different philosophies of risk tolerance, each carrying distinct maternal and fetal consequence profiles.

### 4.2. The 20 mmHg Treatment Gap: Clinical Impact of Threshold Discordance by a Case‐Based Illustration

**FIGURE 2:**
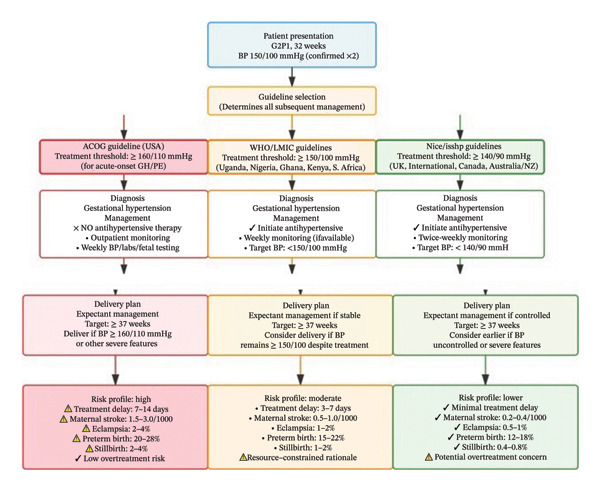
Divergent clinical management pathways based on guideline selection. The figure presents a hypothetical clinical scenario to illustrate how guideline choice may alter management for the same patient presentation. The risk‐profile values are illustrative and should not be interpreted as pooled effect estimates, validated prediction probabilities, or direct estimates from a single study. They summarize the expected direction of risk under different treatment‐threshold strategies using the guideline thresholds in Table 1 and published evidence regarding progression to severe hypertension, maternal morbidity, and potential fetal‐growth concerns. The figure is, therefore, intended as a clinical interpretation aid rather than a quantitative risk‐prediction tool.

To demonstrate the clinical consequences of guideline divergence, we present a hypothetical, representative scenario falling within the contested 20 mmHg treatment gap (Figure 2).

A 28‐year‐old woman (G2P1) presented at 32 weeks’ gestation with a BP of 150/100 mmHg, confirmed four hours later using a standardized technique (seated position after 5 min of rest, validated automated device with appropriately sized cuff at heart level, and Korotkoff phase V for diastolic reading). She remained asymptomatic with no headaches, visual disturbances, or epigastric pain. Laboratory parameters were normal: platelets 220,000/μL, AST 28 IU/L, ALT 24 IU/L, creatinine 0.7 mg/dL, and urine protein–creatinine ratio < 0.3 mg/dL. Fetal assessment revealed appropriate growth, normal amniotic fluid, and reassuring umbilical artery Doppler. All guidelines classify this presentation as gestational hypertension without severe features, yet management diverges fundamentally based on guideline selection.

Under ACOG (2020) protocols, no antihypertensive medication would be initiated because BP remains below the 160/110 mmHg acute‐treatment threshold [3, 11]. Management consists of outpatient monitoring with weekly BP and laboratory assessment, twice‐weekly fetal surveillance, and delivery planned at ≥ 37 weeks unless progression occurs. This approach prioritizes avoiding medication exposure when BP remains in the nonsevere range, reflecting concerns about potential uteroplacental compromise from aggressive BP lowering.

NICE (2023) mandates immediate antihypertensive therapy at BP ≥ 140/90 mmHg for any hypertensive disorder [30]. This patient would commence oral labetalol 100 mg twice daily, titrated to target a BP of ≤ 135/85 mmHg, with twice‐weekly monitoring. This rationale emphasizes maternal cerebrovascular protection, as BP elevations below 160/110 mmHg are associated with progression to hypertensive crisis and stroke [4].

The WHO (2020) and the Uganda Ministry of Health (2024) adopt an intermediate position, initiating treatment at ≥ 150/100 mmHg, precisely this patient’s BP [23]. This threshold reflects pragmatic adaptation to resource‐limited contexts where delayed presentation, limited monitoring capacity, and restricted stroke treatment access necessitate earlier intervention than ACOG recommends, while avoiding NICE’s lower threshold due to medication availability constraints [2, 8].

Figure 2 illustrates the resulting management divergence: the same patient receives observation only (ACOG), immediate treatment initiation (WHO/Uganda Ministry of Health), or treatment that should have started at an earlier visit when BP first exceeded 140/90 mmHg (NICE/ISSHP). This exemplifies guideline‐dependent practice where therapeutic decisions reflect institutional guideline adoption rather than patient‐specific risk factors.

The fundamental clinical question remains incompletely resolved: does early intervention at 140–159/90–109 mmHg prevent progression to severe disease and maternal complications or does it expose women to unnecessary medication with potential fetal effects? Most trials have focused on surrogate endpoints rather than rare catastrophic outcomes [31, 32]. While the CHAP trial demonstrated benefit from treating chronic hypertension at ≥ 140/90 mmHg [33], whether these findings apply to gestational hypertension, a condition with distinct pathophysiology involving acute endothelial dysfunction, remains uncertain [3, 4].

From a QI perspective, this case demonstrates that guideline discordance creates a structural barrier to equitable care. Pregnant women with identical presentations receive fundamentally different management based on geographic location or institutional protocol factors unrelated to individual risk. In settings where multiple guidelines circulate without a single adopted standard, this discordance produces provider confusion and inconsistent care pathways [13, 34]. Resolution requires definitive trial evidence comparing maternal and fetal outcomes under different threshold approaches in pregnancy‐specific hypertensive disorders, rather than extrapolating from chronic hypertension data.

The most consequential divergence identified in our analysis is the treatment initiation threshold ranging from 140 to 160 mmHg SBP across guidelines. This reflects unresolved tension between two competing physiological concerns. Guidelines advocating treatment at ≥ 140/90 mmHg prioritize maternal cerebrovascular protection, citing evidence that even BP elevations below the traditional “severe” threshold are associated with increased stroke risk, progression to hypertensive crisis, and postpartum hemorrhage [3, 4]. The CHAP trial, published after many current guidelines were finalized, provided randomized evidence supporting treatment at ≥ 140/90 mmHg, demonstrating reduced progression to preeclampsia with severe features and indicated preterm birth without increased small‐for‐gestational‐age infants when mild chronic hypertension was actively treated [4, 33].

Conversely, ACOG’s more conservative threshold of ≥ 160/110 mmHg for acute treatment in gestational hypertension and preeclampsia reflects concern that aggressive BP lowering may compromise uteroplacental perfusion in a vascular bed already characterized by inadequate spiral artery remodeling and increased resistance [11]. This physiological concern has theoretical merit but lacks definitive clinical trial evidence demonstrating harm from treating BP in the 140–159/90–109 mmHg range during pregnancy.

The evidence base supporting either approach remains incomplete. Most randomized trials of antihypertensive therapy in pregnancy have been insufficiently powered to detect differences in rare but catastrophic maternal outcomes such as stroke or eclampsia, instead focusing on surrogate endpoints such as progression to severe hypertension [31, 32]. Observational data consistently demonstrate that untreated BP ≥ 150/100 mmHg is associated with increased maternal morbidity, yet randomized trial evidence confirming benefit from treatment at 140–149/90–99 mmHg emerged only recently [33]. Our analysis reveals that LMIC guidelines have pragmatically adopted an intermediate threshold (≥ 150/100 mmHg), potentially representing a reasonable compromise that initiates treatment before BP reaches the highest‐risk range, while avoiding medication exposure at levels where evidence of benefit remains debated.

### 4.3. Misclassification as a Mechanism of Disparate Care

Our finding that identical clinical presentations are classified differently depending on guideline choice has profound implications beyond epidemiological reporting. A pregnant woman presenting with new‐onset hypertension and FGR will be diagnosed with preeclampsia under NICE, ISSHP, or SOGC criteria, triggering heightened surveillance, earlier consideration of magnesium sulfate prophylaxis, and more aggressive delivery planning [7, 15]. Under ACOG criteria, this same patient receives a diagnosis of gestational hypertension complicated by FGR, a classification that may not prompt the same urgency of intervention despite identical maternal BP and fetal compromise [11].

The clinical relevance of this reclassification extends beyond semantics. Retrospective cohort studies have demonstrated that pregnancies meeting the NICE/ISSHP preeclampsia criteria, but not the ACOG criteria, nonetheless experience elevated rates of adverse maternal outcomes compared to those with uncomplicated gestational hypertension, suggesting that the broader diagnostic criteria may identify a genuinely higher‐risk population rather than simply inflating prevalence through definitional expansion [13, 14]. However, whether applying the preeclampsia label and its associated interventions to this population improves outcomes remains unproven in prospective trials.

### 4.4. Implementation Challenges in Low‐Resource Settings: When Guideline Discordance Meets System Constraints

Our analysis revealed that guideline complexity disproportionately impacts LMIC healthcare systems, where multiple international guidelines circulate without a single adopted national standard [4, 23]. Provider confusion documented in qualitative studies is not merely an educational gap but a structural problem: when ACOG, NICE, WHO, and local ministry of health protocols provide conflicting recommendations for the same clinical scenario, evidence‐based practice becomes impossible, even for highly trained clinicians [13, 34].

This confusion is compounded by resource constraints that make adherence to any single guideline difficult. Validated automated BP devices recommended by ISSHP and FIGO for accurate measurement remain unavailable in many facilities, forcing reliance on aneroid devices of unknown calibration status [2, 7]. Intermittent stock‐outs of first‐line antihypertensives and magnesium sulfate force clinicians to ration treatment, effectively raising the de facto threshold for intervention regardless of which guideline is followed [8, 9]. Poor antenatal care attendance in many LMICs means that hypertension is often first detected at advanced gestational age or in crisis, eliminating the opportunity for the graded, threshold‐based management that guidelines assume [17].

Our case scenario illustrated these challenges: a woman presenting at 32 weeks with a BP of 150/100 mmHg requires immediate antihypertensive therapy under NICE guidelines, whereas treatment “should be considered” under WHO guidance, and observation only is recommended under ACOG protocols. A facility following NICE but lacking medication stock will fail to meet the standard; a facility following ACOG provides observation consistent with that guideline but potentially exposes the woman to progression to severe hypertension. Neither represents guideline failure. However, both represent guideline discordance creating impossible clinical situations.

### 4.5. Measurement Variability as Hidden Contributor to Threshold Confusion

While most attention has focused on numerical threshold differences, our analysis identified substantial variation in recommended BP measurement techniques across guidelines. These technical specifications are not interchangeable: systematic measurement differences of 5–10 mmHg can result from positional changes, inadequate rest periods, or improper cuff size—differences sufficient to reclassify a patient from one management category to another when thresholds are tightly defined [29].

Validation of BP devices in pregnancy receives variable emphasis across guidelines, yet nonpregnant validation does not ensure accuracy in preeclampsia due to the characteristic high systemic vascular resistance and increased arterial stiffness [7]. Many automated devices underestimate BP in severe preeclampsia, potentially delaying recognition of severe‐range hypertension even when measurement is frequent. LMIC guidelines acknowledge this reality by permitting aneroid devices when automated alternatives are unavailable, introducing a pragmatic exception that may paradoxically improve detection if aneroid measurement is performed more carefully than rushed automated measurement [2].

### 4.6. Toward Evidence‐Based Threshold Harmonization

Resolution of guideline discordance requires confronting the reality that key questions remain unanswered by high‐quality randomized trial evidence. Specifically,

1. What is the optimal BP treatment threshold in gestational hypertension and preeclampsia to minimize both maternal cerebrovascular risk and uteroplacental insufficiency? The CHAP trial addressed this question for chronic hypertension, demonstrating benefit from treatment at ≥ 140/90 mmHg [33], but similar evidence for gestational hypertension and preeclampsia remains absent.
2. What BP target range optimizes maternal and fetal outcomes? Current targets range from < 160/110 mmHg, ≤ 135/85 mmHg, to 110–140/< 85 mmHg. Observational data suggest that DBP < 80 mmHg is associated with increased small‐for‐gestational‐age infants [31], but whether this represents causation or confounding by disease severity is unclear.
3. Should fetal/placental dysfunction (FGR, abnormal Doppler) be included as a preeclampsia diagnostic criterion or classified separately as gestational hypertension with placental insufficiency? This question has pathophysiological, epidemiological, and clinical management implications that remain contested [7, 14].
4. Can risk stratification tools identify which women with BP 140–159/90–109 mmHg require immediate treatment versus expectant management? Biomarkers such as sFlt‐1/PlGF ratio or integration of clinical features into predictive algorithms may enable individualized threshold decisions [35], but prospective validation demonstrating improved outcomes from biomarker‐guided threshold decisions is lacking.

Recent evidence also supports the potential value of inexpensive inflammatory and lipid‐related markers in HDP risk stratification, particularly in settings where angiogenic biomarker testing is unavailable. Bulu and Bulu reported that the neutrophil‐to‐high‐density lipoprotein cholesterol ratio (NHR) was significantly higher among women with gestational hypertension and preeclampsia than among normotensive controls and that NHR, C‐reactive protein, and platelet count were independent predictors of gestational hypertension and preeclampsia [36]. This finding strengthens the biological plausibility of integrating inflammatory and cardiometabolic markers with BP thresholds for disease‐severity assessment. However, because the study was retrospective and cross‐sectional, these markers should be considered supportive risk‐stratification tools rather than replacements for guideline‐based BP thresholds, and prospective validation is needed before they can inform treatment‐initiation thresholds or delivery decisions.

In the absence of definitive trial evidence, consensus‐building must balance maternal cerebrovascular protection (favoring lower thresholds) against concerns about fetal growth and iatrogenic prematurity (favoring higher thresholds), while acknowledging that different healthcare contexts may reasonably prioritize these competing risks differently. LMIC settings with high maternal mortality and limited stroke treatment capacity may rationally adopt lower treatment thresholds even if this modestly increases small‐for‐gestational‐age rates, whereas high‐income settings with robust acute stroke care may tolerate slightly higher BP to minimize neonatal intensive care admissions.

### 4.7. Implications for Practice and Policy

Clinicians working with multiple guidelines should use one institutional protocol to ensure consistent management and regularly audit outcomes. Where no local protocol exists, contemporary international guidance such as ISSHP (2022) and NICE (2023) offer the most up‐to‐date evidence. In LMIC settings, a threshold of ≥ 150/100 mmHg is a practical balance between maternal benefit and limited resources. For women within the 140–159/90–109 mmHg “treatment‐gap” range, shared decision‐making is essential to address evidence uncertainty and patient preferences. Above all, accurate BP measurement should take priority over small threshold differences.

Guideline developers should work toward harmonizing core thresholds, especially treatment initiation and severe hypertension definitions, while allowing contextual flexibility. Guidelines must clearly indicate where recommendations are based on strong evidence versus expert consensus. Policymakers should invest in LMIC implementation trials to determine optimal thresholds in real‐world settings. Developing simple, point‐of‐care clinical decision tools is crucial to support rapid and appropriate management in resource‐limited environments.

### 4.8. Limitations

Several limitations merit consideration. As a narrative review, this study may be subject to selection bias and heterogeneity in the included evidence. Our analysis is descriptive and threshold‐focused; we did not systematically assess quality of evidence supporting each guideline’s recommendation or grade recommendations using GRADE methodology. A systematic review of primary trial evidence was not performed. Some national guidelines lack transparent methodological documentation, limiting quality appraisal. We also did not examine implementation strategies, guideline dissemination approaches, or adherence measurement across context factors that may be as important as threshold selection in determining real‐world outcomes. While our case scenario is clinically realistic, it is hypothetical: actual management decisions incorporate clinical judgment, shared decision‐making, and contextual factors not captured in protocol‐driven scenario analysis. Guidelines are periodically updated, and our analysis reflects current versions as of 2024, but some may have been superseded by the time of publication. Variability in guideline publication years (e.g., WHO 2011 still in active use) may introduce temporal inconsistencies, although inclusion was justified based on current applicability. Despite these limitations, methodological transparency and multisource triangulation were used to mitigate bias.

## 5. Conclusion

HDP represent a global health priority, yet international guidelines that should provide clarity instead offer conflicting directions, at precisely the decision points where evidence‐based guidance is most needed. The 20 mmHg treatment threshold variation documented in this analysis is not a trivial discrepancy, but a fundamental disagreement about when pharmacological intervention is warranted, a decision with direct implications for maternal stroke risk, fetal growth, and healthcare resource utilization.

Until definitive trial evidence resolves these questions, guideline harmonization must be driven by consensus‐building processes that transparently acknowledge evidence gaps, explicitly weigh competing maternal and fetal risks, and tailor recommendations to implementation context. For the pregnant woman presenting with a BP of 150/100 mmHg at 32 weeks’ gestation, the question of whether treatment should be initiated should have an evidence‐based answer that does not depend on which guideline her provider happens to follow. Achieving that goal requires not only better evidence but also the professional courage to adopt unified international standards even in the face of residual uncertainty.

## Funding

The authors did not receive any funding for the publication of this manuscript.

## Ethics Statement

The authors have nothing to report.

## Consent

The authors have nothing to report.

## Conflicts of Interest

The authors declare no conflicts of interest.

## Data Availability

Data sharing is not applicable to this article as no datasets were generated or analyzed during the current study.
